# A controlled cross-over study to evaluate the efficacy of improvised dry and wet emergency decontamination protocols for chemical incidents

**DOI:** 10.1371/journal.pone.0239845

**Published:** 2020-11-04

**Authors:** Felicity Southworth, Thomas James, Louise Davidson, Natalie Williams, Thomas Finnie, Tim Marczylo, Samuel Collins, Richard Amlôt

**Affiliations:** 1 Emergency Response Department Science & Technology, Health Protection Directorate, Public Health England, London, United Kingdom; 2 Chemicals and Environmental Effects Department, Centre for Radiation, Chemicals and Environmental Hazards, Public Health England, Didcot, Oxfordshire, United Kingdom; 3 Toxicology Department, Centre for Radiation, Chemicals and Environmental Hazards, Public Health England, Didcot, Oxfordshire, United Kingdom; University of Birmingham College of Medical and Dental Sciences, UNITED KINGDOM

## Abstract

The UK Initial Operational Response (IOR) to chemical incidents includes improvised decontamination procedures, which use readily available materials to rapidly reduce risk to potentially exposed persons. A controlled, cross-over human volunteer study was conducted to investigate the effectiveness of improvised dry and wet decontamination procedures on skin, both alone, and in sequence. A simulant contaminant, methyl salicylate (MeS) in vegetable oil with a fluorophore was applied to three locations (shoulder, leg, arm). Participants then received no decontamination (control) or attempted to remove the simulant using one of three improvised protocols (dry decontamination; wet decontamination; combined dry and wet decontamination). Simulant remaining on the skin following decontamination was quantified using both Gas Chromatography Tandem Mass Spectrometry (GC-MSMS) for analysis of MeS and UV imaging to detect fluorophores. Additionally, urine samples were collected for 24 hours following application for analysis of MeS. Significantly less simulant was recovered from skin following each improvised decontamination protocol, compared to the no decontamination control. Further, combined dry and wet decontamination resulted in lower recovery of simulant when compared to either dry or wet decontamination alone. Irrespective of decontamination protocol, significantly more simulant remained on the shoulders compared to either the arms or legs, suggesting that improvised decontamination procedures are less effective for difficult to reach areas of the body. There was no effect of decontamination on excreted MeS in urine over 24 hours. Overall, findings indicate that improvised decontamination is an effective means of rapidly removing contaminants from skin, and combinations of improvised approaches can increase effectiveness in the early stages of decontamination and in the absence of specialist resources at an incident scene. However, the variable control and consistency of improvised decontamination techniques means that further intervention is likely to be needed, particularly for less accessible areas of the body.

## Introduction

In the event of a chemical incident, rapid decontamination of the people exposed is essential to minimise injury, prevent loss of life, and inhibit the uncontrolled spread of contamination. In recent years, UK mass casualty decontamination protocols have been enhanced through the introduction of the Initial Operational Response (IOR) programme [1]. The purpose of IOR is to equip non-specialist frontline responders with strategies to rapidly reduce the risk to potentially contaminated people, via: evacuation from hazardous areas, removal of contaminated clothing, and improvised dry and wet decontamination protocols using any available materials.

Improvised decontamination involves dry and/or wet procedures to remove contaminants from the body, using readily available materials [1]. According to IOR guidance, dry decontamination should be conducted in the first instance where a non-caustic chemical is suspected then potentially followed by improvised wet decontamination. If signs and symptoms of a caustic chemical are apparent, improvised wet decontamination should be conducted as the default measure. Dry decontamination involves using a dry absorbent material (e.g., tissue) to blot and rub the body. Improvised wet decontamination can be administered using any available water source, typically following a ‘rinse-wipe-rinse’ (RWR) procedure [1]. Improvised decontamination may then be followed by interim wet decontamination set-up using Fire and Rescue Service (FRS) frontline equipment, followed by structured mass decontamination showering following the arrival of specialist decontamination units [1].

Whilst interim decontamination showers are a relatively rapid means of administering emergency decontamination, they will usually not be available until a minimum of 25 minutes after the reporting of an incident. Improvised decontamination procedures on the other hand, can be initiated as soon as emergency responders arrive on the scene, usually within 15 minutes, or potentially even sooner by members of the public. Since some hazardous chemicals can have adverse effects within minutes of exposure [2], and systemic exposure levels will be dependent upon the length of time a chemical remains on the skin, improvised decontamination is a crucial step in harm reduction in a chemical incident.

Whilst decontamination of skin using IOR methods has been subjected to controlled efficacy testing *in vitro* [3, 4] little is known of the efficacy of these individual decontamination procedures when conducted in sequence. Although human volunteer studies have investigated the effectiveness of dry decontamination [5–7], improvised wet decontamination has yet to be investigated. Further, as mass decontamination strategies rely on implementation in series of dry followed by wet decontamination protocols as new resources become available at the scene of the incident, it is reasonable to explore the potential beneficial effects of sequential improvised dry and wet decontamination protocols, before these resources arrive.

To evaluate the effectiveness of decontamination protocols in human volunteer trials, simulant contaminants which can be safely applied to human volunteers are required. Simulant contaminants should mimic the physicochemical properties of more harmful chemicals, whilst remaining non-toxic at the applied dose. Many previous human volunteer studies have employed methyl salicylate (MeS), a simulant for sulphur mustard with a similar vapour pressure, water solubility and biological half-life, but sufficiently low toxicity to be safe for dermal application to humans [8].

To date, most studies of decontamination efficacy have focused on the amount of simulant remaining on the skin [5–7]. Whilst this is important for blister agents and preventing the uncontrolled spread of contaminants, for many hazardous chemicals the systemic availability is more important for predicting potential health consequences. Ideally, this would mean measurement of simulant in blood, however levels excreted in urine can be employed as a surrogate for systemic exposure.

The primary aim of the present human volunteer study was to determine the effectiveness of improvised dry and wet decontamination procedures, both alone and in combination, for removal of MeS from skin and reducing MeS excreted in urine. A secondary aim of the study was to determine decontamination efficacy at sites (arm, leg, shoulder) with differing degrees of accessibility. We hypothesise that combined decontamination will be more effective than individual dry or wet decontamination and that all methods will be less effective in less accessible sites, as determined via the level of recovery of residual simulant from the skin and in 24-hour urine samples post-decontamination.

## Methods

### Participants & study design

Twelve healthy adults (4 females, 8 males; ages 26–62 years) completed a controlled, cross-over study during which they completed four decontamination conditions in a randomised order. Study sessions were separated by a minimum of 4 days. Participant eligibility criteria were developed taking into account participant safety, methodological and ethical considerations. Written informed consent was obtained from all participants on entry to the trial. Ethical approval was independently granted by Public Health England’s Research Ethics and Governance Group.

### Decontamination conditions

Four decontamination conditions were tested in this study; control (A), improvised dry (B), improvised wet (C) and combined dry and wet (D). Participants in the control condition did not undergo any form of decontamination but were asked to stand for the duration of the study and were moved to the different, pre-defined decontamination areas to replicate the movement of volunteers’ in the other decontamination conditions. Conditions B, C and D were conducted according to the protocols outlined in Table 1. Descriptive statistics of decontamination condition characteristic variables are presented in Table 2.

**Table 1:** Decontamination conditions.

Decontamination condition	Protocol description
Improvised dry	This condition followed the IOR dry decontamination procedure [1] with the following modifications:
	Pieces of white roll (Hygiene Rolls White 2-Ply 25cm x 25cm, Tower Supplies, Poole UK) were individually folded in half twice. Participants were instructed to use one piece of white roll at a time and to blot and rub their skin working down the body from the shoulders. Participants were reminded not to come back up the body using the same piece of white roll. Participants were given a total of three minutes but were instructed to continue until they felt they had finished. Participants were free to use as many pieces of white roll as required.
Improvised wet	This condition followed the IOR rinse-wipe-rinse procedure [1].
	Three buckets containing 5L of ambient temperature water were provided, with one bucket containing 0.5% detergent solution (Fairy Liquid, Procter and Gamble, UK). Decontamination lasted a total of 3 minutes. In three stages lasting 1 minute each participants were instructed to:
	… Rinse the body using clean water and a 1L jug provided
	… Wipe themselves down using a sponge and the detergent solution, in a downward motion from the shoulders, not returning up the body
	… Rinse for a final time using clean water and the 1L jug
Combined	Improvised dry and improvised wet decontamination performed in sequence.

**Table 2:** Mean (*SD*) and range for decontamination condition variables.

	A–Control	B–Dry	C–Wet	D–Combined
Ambient temperature (°C)	18.3 (*1*.*87*), 15.5–21.1	19.1 (*1*.*65*), 16.3–21.6	19.5 (*2*.*02*), 16.0–22.4	19.2 (*1*.*25*), 16.9–20.8
Quantity of white roll (no. sheets)	-	8 (*6*.*11*), 1–20	-	7 (*4*.*55*), 2–17
Dry decontamination time (min:sec)	-	2:25 (*0*:*19*), 1:58–2:44	-	2:08 (*0*:*36*), 0:55–2:53
Water temperature (°C)	-	-	18.9 (*1*.*81*), 15.3–23.9	18.5 (*2*.*33*), 15.7–24.7

### Study procedure

**Fig 1:**
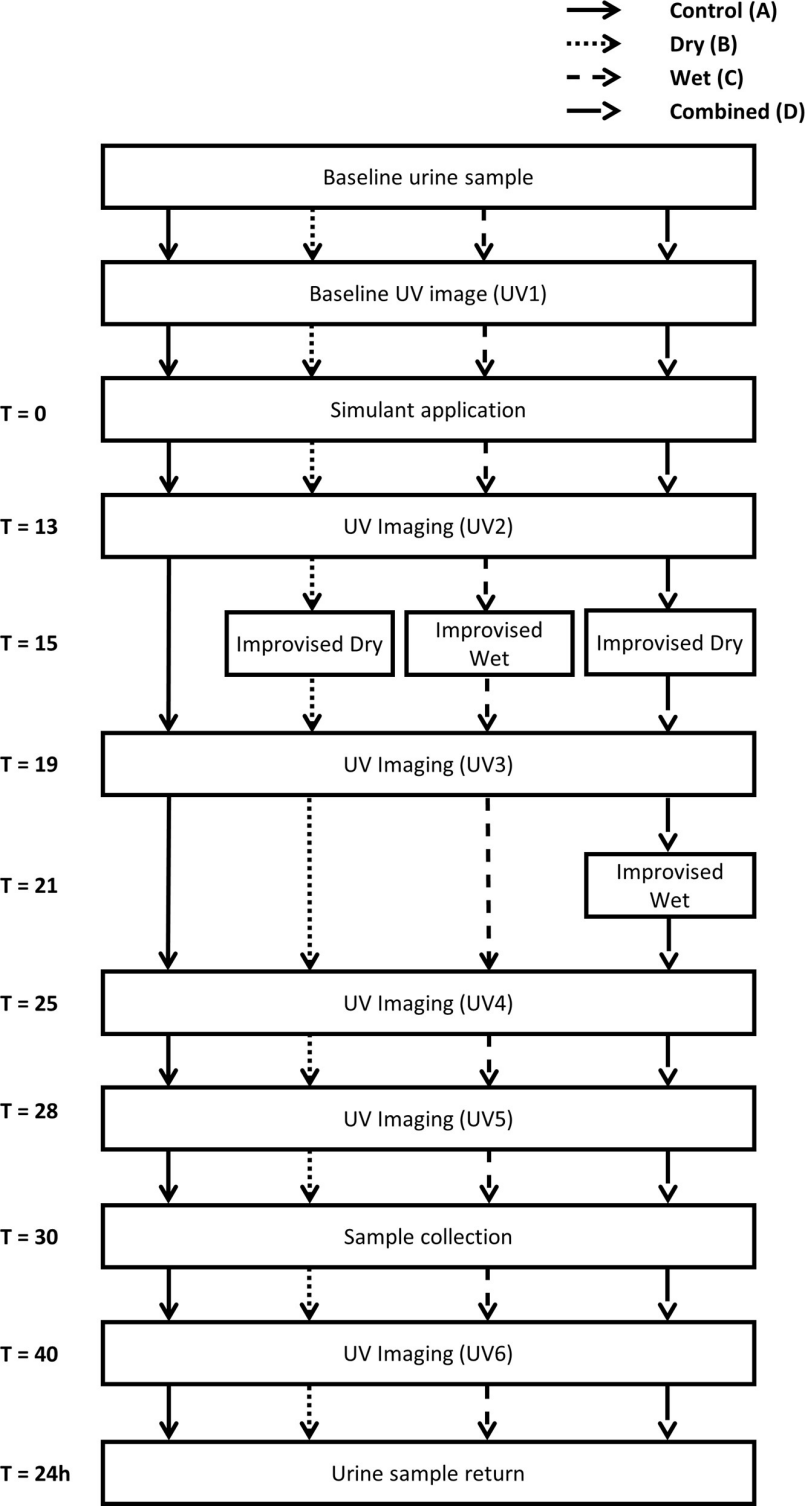
Schematic representation of the trial protocol. T = time in minutes.

Fig 1 shows the timeline for each stage of the study procedure in each decontamination condition.

#### Baseline urine collection

Participants were provided with a list of products containing MeS, and instructed not to use or consume these products for 24 hours before the study session. Participants were also instructed not to shower or wash within 2 hours prior to the study session. On arrival participants’ adherence to pre-study instructions was assessed using a brief interview, along with checks for any in medical history that may impact their eligibility. Prior to study commencement, participants supplied a baseline urine sample (10–50 mL) in a 50 mL Falcon tube (Fisher Scientific, UK). The total volume of urine excreted was recorded. Urine samples were immediately stored at 4°C.

Participants then changed into black or dark blue polyester/nylon swim wear provided, and a baseline UV image (UV1) was then captured.

#### Simulant application

The simulant consisted of a 1:1 (v/v) mixture of MeS (purity >99%, Fisher Scientific, UK) and vegetable oil (100% rapeseed oil, The Co-operative, UK) containing 4 mg/mL Invisible Red S (IRS; Apex, Ely, UK), a UV fluorescent compound. Vegetable oil was added to increase the persistence of the simulant due to decreased MeS volatilisation.

**Fig 2:**
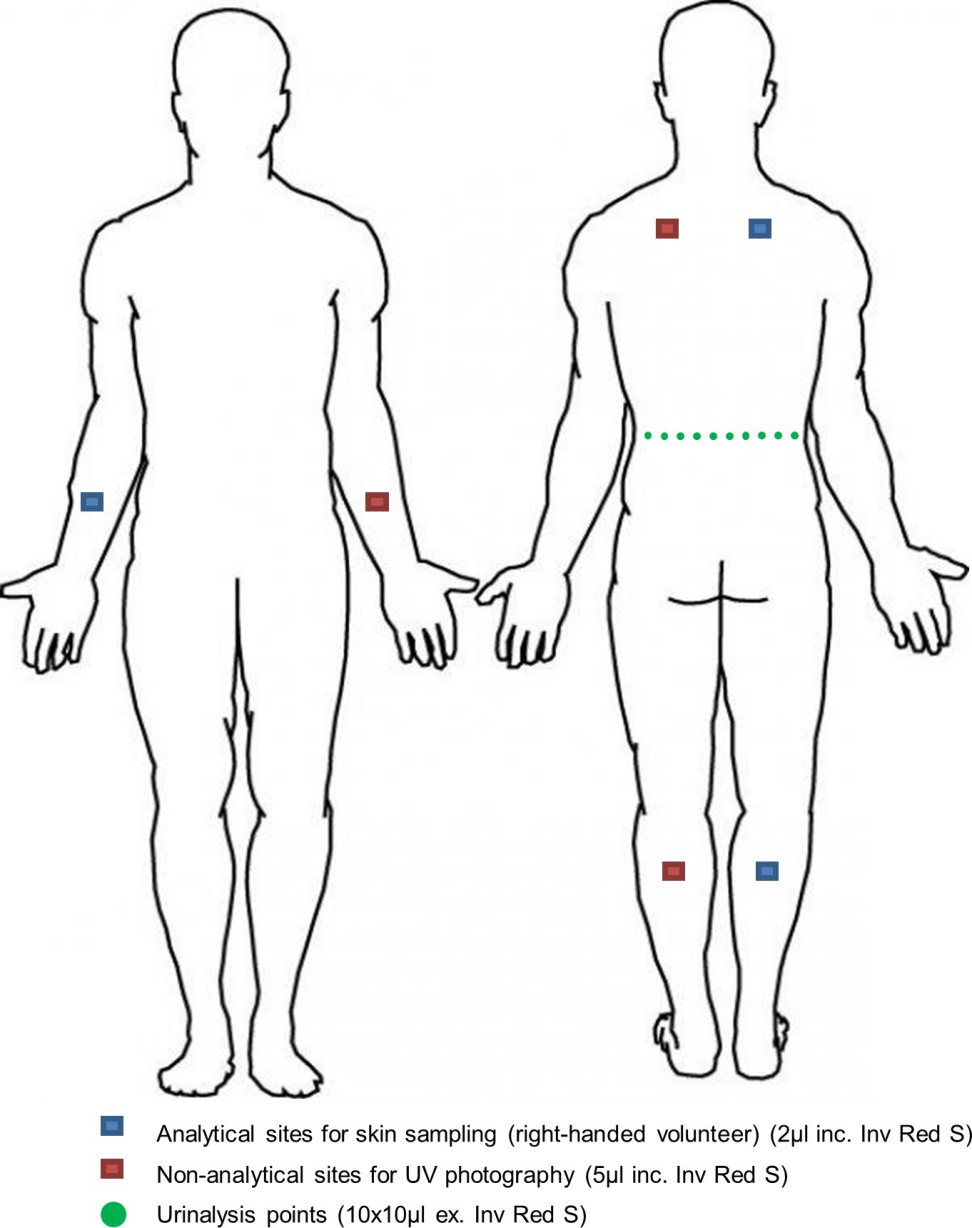
Schematic representation of simulant application sites.

Participants were instructed to lie face-down on a table with palms facing up. Simulant was applied to specific areas of the shoulder, forearm and calf, chosen to represent hard, medium and easy areas to decontaminate, respectively. The analytical application sites for skin sampling were added to the self-declared dominant side of the body. Application sites were pre-marked using an acrylic template and indelible ink. The non-analytical sites were used for UV image analysis. Simulant was applied to each of the analytical and non-analytical sites (2 μL and 5 μL respectively, total 21 μL; Fig 2) using a positive displacement pipette. To increase the applied dose to facilitate the detection of MeS in urine an additional two rows of 10 x 10 μL of simulant without IRS was applied across the back (lower back for male participants, middle back for female participants). Participants were instructed to avoid touching the application sites when not actively decontaminating, to reduce removal or transfer of the simulant to other parts of the body. A post-simulant application UV image (UV2) was taken at thirteen minutes.

#### Decontamination

**Fig 3:**
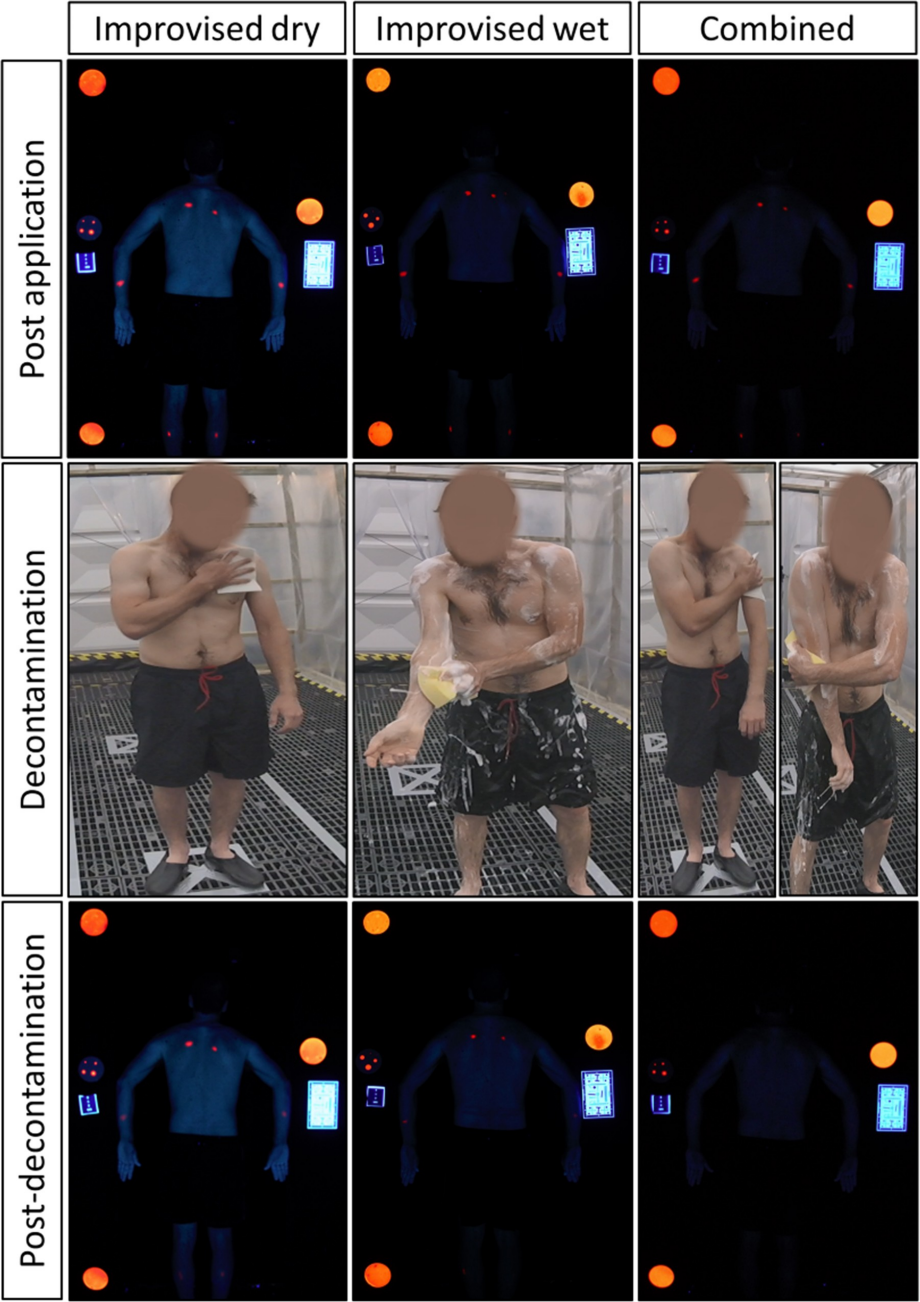
A participant performing improvised dry, improvised wet and the combined decontamination conditions together with representative UV images captured post MeS application and post decontamination. The red areas of fluorescence on the skin of the participant represent areas of MeS contamination. Area (mid left of images) and emittance (top left, bottom left and mid right of images) MeS calibration disks are also shown.

Decontamination (conditions B, C and D; Fig 3) began at 15 minutes post simulant application to replicate the guidance time for decontamination initiation in IOR guidance [1]. In the combined condition (D), the second decontamination protocol began at 21 minutes post simulant application. Although the simulant was only applied to specific areas, participants were instructed to decontaminate as if they had been contaminated all over their body. UV photography and sample collection timings occurred according to the procedure outlined in Fig 1.

#### Skin sample collection

Following decontamination procedures, volunteers returned to the application table. Skin samples were taken from the analytical application sites (see Fig 2) using tape-strip sampling 30 minutes after simulant application. Six 22 mm D-Squame adhesive discs (Cuderm Corporation, USA) were applied sequentially to each site to remove upper layers of the stratum corneum. The adhesive discs were applied using forceps and uniform pressure was applied to each disc using a D-Squame applicator (Cuderm Corporation, USA). Forceps were used to remove each disc and place them into 1 of 3 pre-filled glass vials containing 10 mL of dichloromethane (DCM, Fisher Scientific), (Vial A—discs one and two; Vial B—discs three and four; Vial C—discs five and six).

Process controls were created by spiking 2 μL of simulant onto two D-Squame discs. One disc was placed into DCM immediately following simulant application to act as a control against theoretical recovery, while the other was placed into a vial of DCM during skin sample collection to normalise against potential evaporative loss of simulant due to ambient conditions.

Samples were stored at 5°C until subsampling. Aliquots (1 mL) were sub-sampled from each sample vial at least 24 hours following sample collection and were coded to sample-blind the analyst.

#### Urine sample collection

Participants collected a sample of urine every time they urinated for 24 hours following simulant application. For each sample, participants recorded the total volume urinated using a 500 mL measuring cylinder and aliquoted up to 50 mL into a falcon tube (Fisher Scientific). Participants recorded the time of each urine sample and provided details of any missed samples. A 2 mL sub-sample was taken from each sample including the baselines and stored in a Cryo.S vial (Greiner Bio-One) at -20°C in a Human Tissue Authority (HTA) compliant freezer prior to analysis.

### Skin and urine sample analysis

Skin sample analysis was conducted by gas chromatography tandem mass spectrometry (GC-MSMS) as outlined by James *et al*. [9] and MeS was extracted from urine samples by supported liquid extraction prior to GC-MSMS as outlined by James *et al* [10].

### UV photography and image analysis

Whole-body UV images were captured at a total of six time points during the study session (Figs 2 and 3) to record the spread and intensity of the simulant on the skin. Images were acquired in a Mobile Image Analysis Unit (MIAU) as previously described [11].

Area and emittance calibration discs were included in each image, positioned in pre-defined locations within the MIAU, such that they were positioned in the same plane as the participants inside the MIAU. Area calibration discs comprised three circular Whatman No. 1 110 mm Filter Papers (GE Healthcare, Little Chalfont, UK) saturated with simulant, and positioned in three pre-defined locations. For the emittance calibration, a series of 1 μL, 2 μL, 5 μL and 10 μL MeS volumes were applied to a Whatman No 1 110 mm Filter Paper. Area calibration discs were replaced weekly and emittance calibration discs daily.

Images were analysed using bespoke software [12], which identified areas of fluorescence in images. Images were segmented to extract clusters of 20 or more red pixels. For each cluster, the number of red pixels and their total intensity was recorded. Area of fluorescence was calculated for each application site by comparing the number of red pixels against the mean number of pixels for the three area calibration discs. For each image, an emittance calibration curve was created using the doses of simulant applied to the emittance calibration disc, using a best-fit linear plot forced through zero (i.e., of the form intensity = a × simulant). An emittance index score was calculated for each application site by comparing the total intensity of red pixels to the emittance calibration curve for that image (i.e., an emittance index score of 5 would indicate equivalent intensity to the 5 μl dose).

### Interpretation and statistics

Outcome measures were analysed using repeated measures analysis of variance (ANOVA), with decontamination condition as an independent variable. Planned contrasts compared the three active decontamination conditions to the no decontamination control, the combined condition to the dry and wet conditions, and the dry to the wet condition. For skin samples and UV images, application site (arm, leg, shoulder) was also included as an independent variable, to investigate differential effects of decontamination protocols for each application site. Planned contrasts compared the shoulder to the arm and leg and compared the arm to the leg. For all outcome measures, Alpha was 0.05, with Huynh-Feldt sphericity corrections applied for repeated measures effects [13]. Analyses were conducted using IBM SPSS Statistics v.25.

## Results

All participants (n = 12, 4 females and 8 males) completed all study conditions. Recorded protocol deviations were reviewed, and 7 participants’ data was removed from urinalysis due to missed samples. Process controls taken during the study showed no cross-contamination or major deviation in conditions between trial days.

### UV imaging

All whole-body UV images yielded data pertaining to simulant area and emittance. Simulant application to all sites was consistent and reproducible with no significant differences in either simulant area (cm^2^), *F*(3,33) = 1.02, *p* = .393, or emittance, *F*(3,33) = 0.69, *p* = .552, for post-application (UV2) images (S1 and S2 Figs and S1 and S2 Tables).

**Fig 4:**
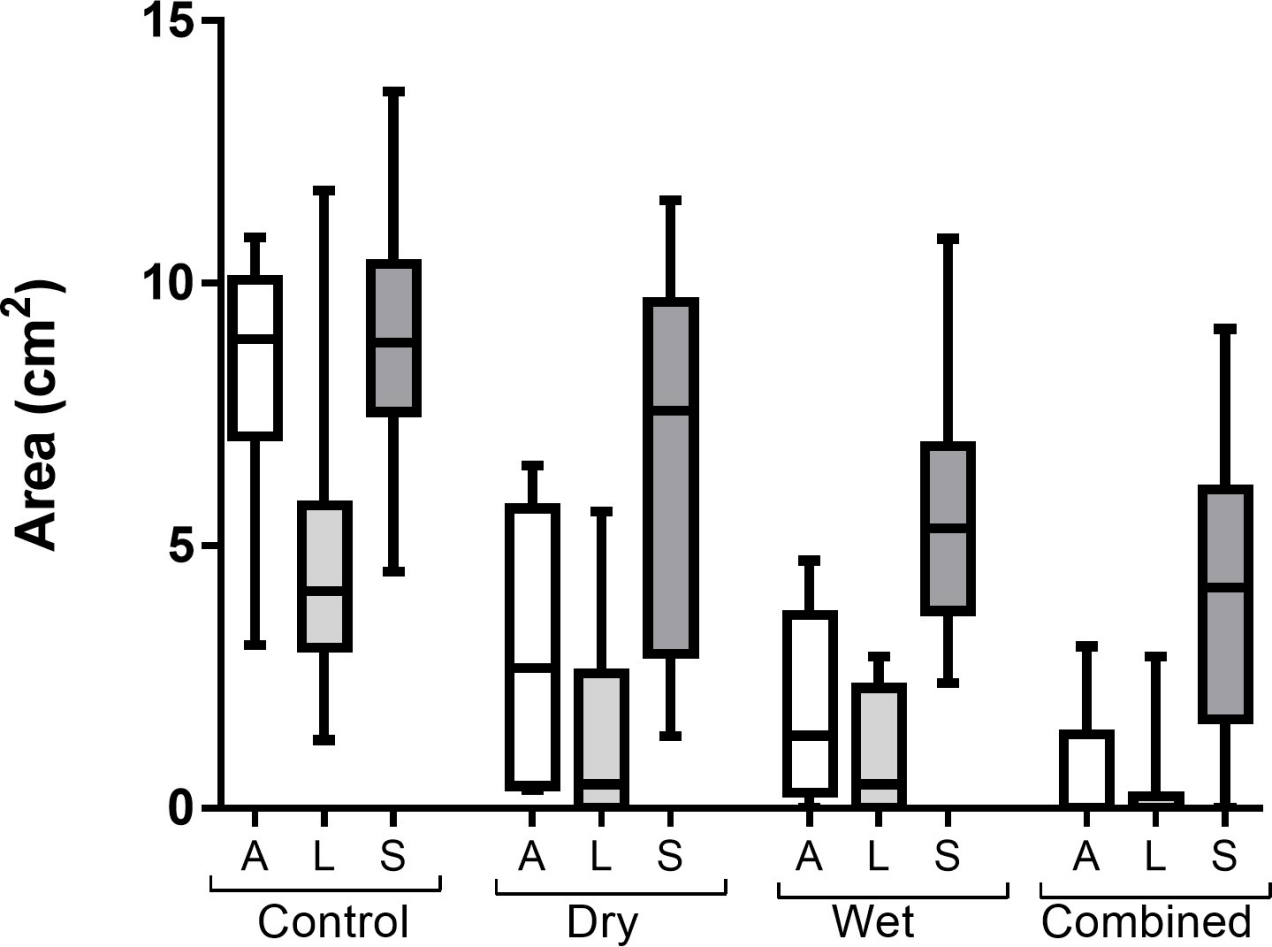
Area of fluorescence post-decontamination (UV4) for each application site in each decontamination condition. Box and whisker plot shows median and inter–quartile range, together with the maximum and minimum values. A = Arm, L = Leg, S = Shoulder.

**Fig 5:**
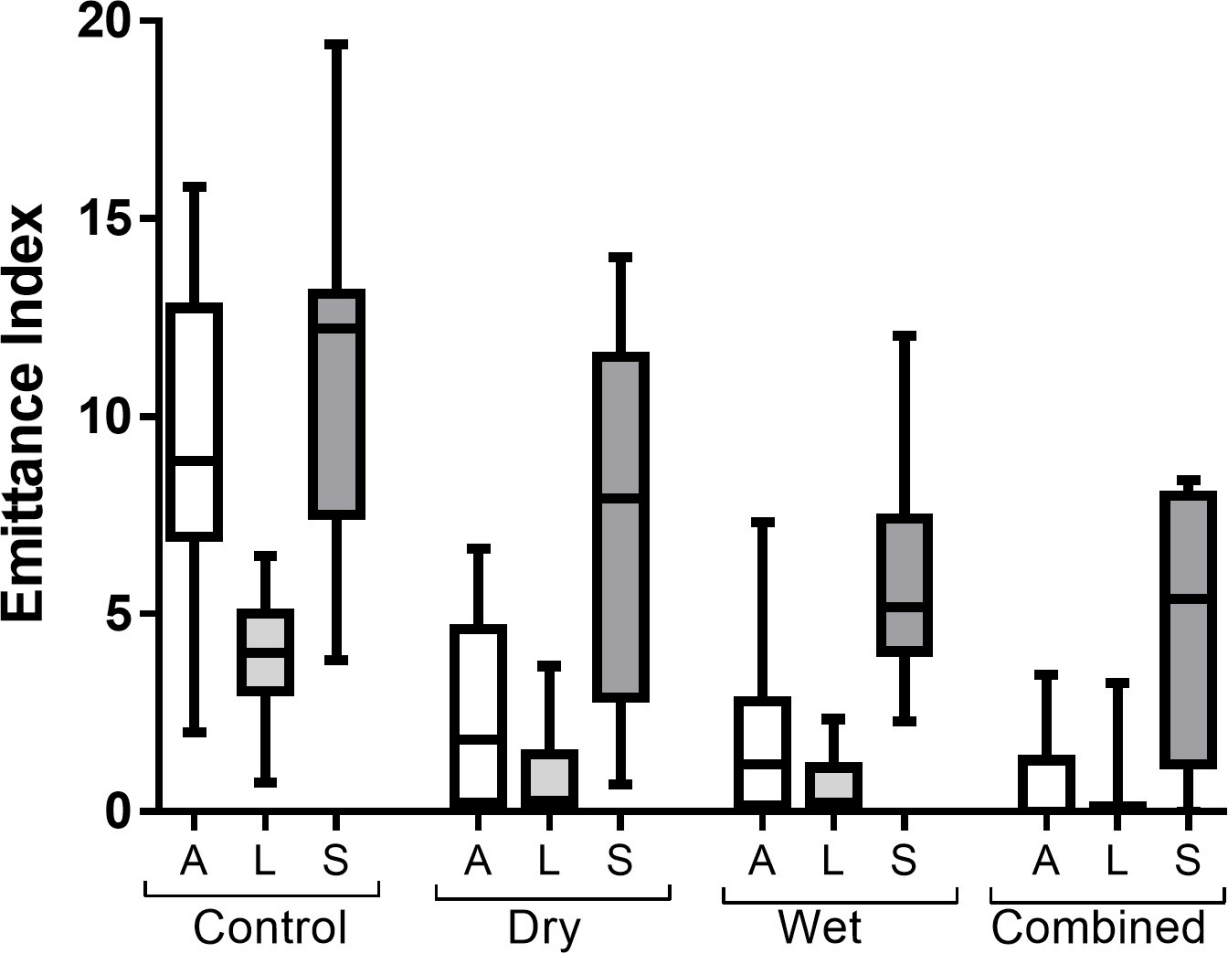
Fluorescent emittance post-decontamination (UV4) for each application site in each decontamination condition. Box and whisker plot shows median and inter–quartile range, together with the maximum and minimum values. A = arm, L = leg, S = shoulder.

Data from post-decontamination (UV4) images showed a significant main-effect of decontamination condition for both area, *F*(3,33) = 53.89, *p* < .001 and emittance *F*(3,33) = 39.34, *p* < .001. Area and emittance were both significantly lower following decontamination compared to no decontamination control, *F*(1,11) = 126.38, *p* < .001 and *F*(1,11) = 59.63, *p* < .001 respectively (Figs 4 and 5 and S1 and S2 and S1 and S2 Tables), and further pairwise comparisons confirmed that both were significantly lower in each of the active decontamination conditions compared to the control (all *p* < .001). Planned contrasts also found that simulant area and emittance were significantly lower in the combined condition compared to the dry and wet conditions alone, *F*(1,11) = 21.48, *p* = .001 and *F*(1,11) = 14.03, *p* = .003, respectively.

Across conditions B, C and D, for post-decontamination (UV4), there was a significant main-effect of application site for both simulant area, *F*(2,22) = 54.35, *p* < .001 and emittance, *F*(2,22) = 44.76, *p* < .001 and no significant interaction between decontamination condition and application site, *F*(4,44) = 0.75, *p* = .540 and *F*(4,44) = 1.46, *p* = .238, respectively. Planned contrasts showed that simulant area and emittance were significantly higher on the shoulder compared to the arm and leg, *F*(1,11) = 74.31, *p* < .001, and *F*(1,11) = 57.86, *p* < .001, respectively (Figs 4 and 5 and S1 and S2 Tables). Furthermore, simulant area and emittance were significantly higher on the arm compared to the leg, *F*(1,11) = 6.85, *p* = .024 and *F*(1,11) = 6.18, *p* = .030, respectively (Figs 4 and 5).

Within the control condition, there was also a significant main-effect of application site on simulant area and emittance, *F*(2,22) = 16.15, *p* < .001 and *F*(2,22) = 22.22, *p* < .001, respectively, although the pattern of distribution of simulant across application sites differed from the active decontamination conditions. Pairwise comparisons found that simulant area and emittance were significantly lower on the leg compared to both the arm and shoulder (all *p* < .01), but that there was no significant difference in simulant area or emittance between the arm and shoulder (*p* = .342 and *p* = .096 respectively). The difference in simulant area and emittance between the arm and the leg significantly reduced in the active decontamination conditions (B, C and D) compared to the control, *F*(1,11) = 11.64, *p* = .006 and *F*(1,11) = 19.22, *p* = .001, respectively. Whilst the difference in simulant area between the shoulder compared to the arm and leg significantly increased in the conditions B, C and D compared to the control, *F*(1,11) = 6.89, *p* = .024, the difference in simulant emittance did not significantly change, *F*(1,11) = 0.54, *p* = .480.

Simulant area and emittance values remained consistent between UV4 and UV5 for all conditions (S1 and S2 Tables) confirming that no protocol deviations, e.g. accidental transfer of simulant from one part of the body to another, had occurred post-decontamination and prior to skin sample collection.

### Skin sample analysis

MeS was detected above the limit of quantitation (LOQ; 0.23 ng/mL) in all skin samples including the lower D-squame disks (5 and 6). In general skin sampling confirmed the data derived from UV imaging.

Statistical analysis showed a significant main-effect of decontamination condition on MeS recovered, *F*(3,33) = 14.74, *p* = .002. All decontamination interventions resulted in significantly lower MeS recovered from the skin of volunteers compared to controls, with mean total recoveries across the application sites of 220.29 μg (SD 168.81) for controls and 34.33 μg (SD 27.95), 34.01 μg (SD 25.09) and 11.22 μg (SD 11.88) for conditions B, C and D, respectively, *F*(1,11) = 15.12, *p* = .003 (S3 Fig and S3 Table). Further pairwise comparisons confirmed that MeS recovery was significantly lower in each of the active decontamination conditions compared to control (all *p* < .01), however no condition resulted in the complete removal of simulant. Planned contrasts showed that the combined decontamination condition (D) was the most efficacious condition, with an approximate 67% less MeS recovered across volunteers compared to both the dry (B) and wet (C) conditions alone, *F*(1,11) = 17.45, *p* = .002. Dry and wet conditions appeared equally efficacious with no significant difference in MeS recovered between these two conditions, *F*(1,11) < 0.01, *p* = .974.

**Fig 6:**
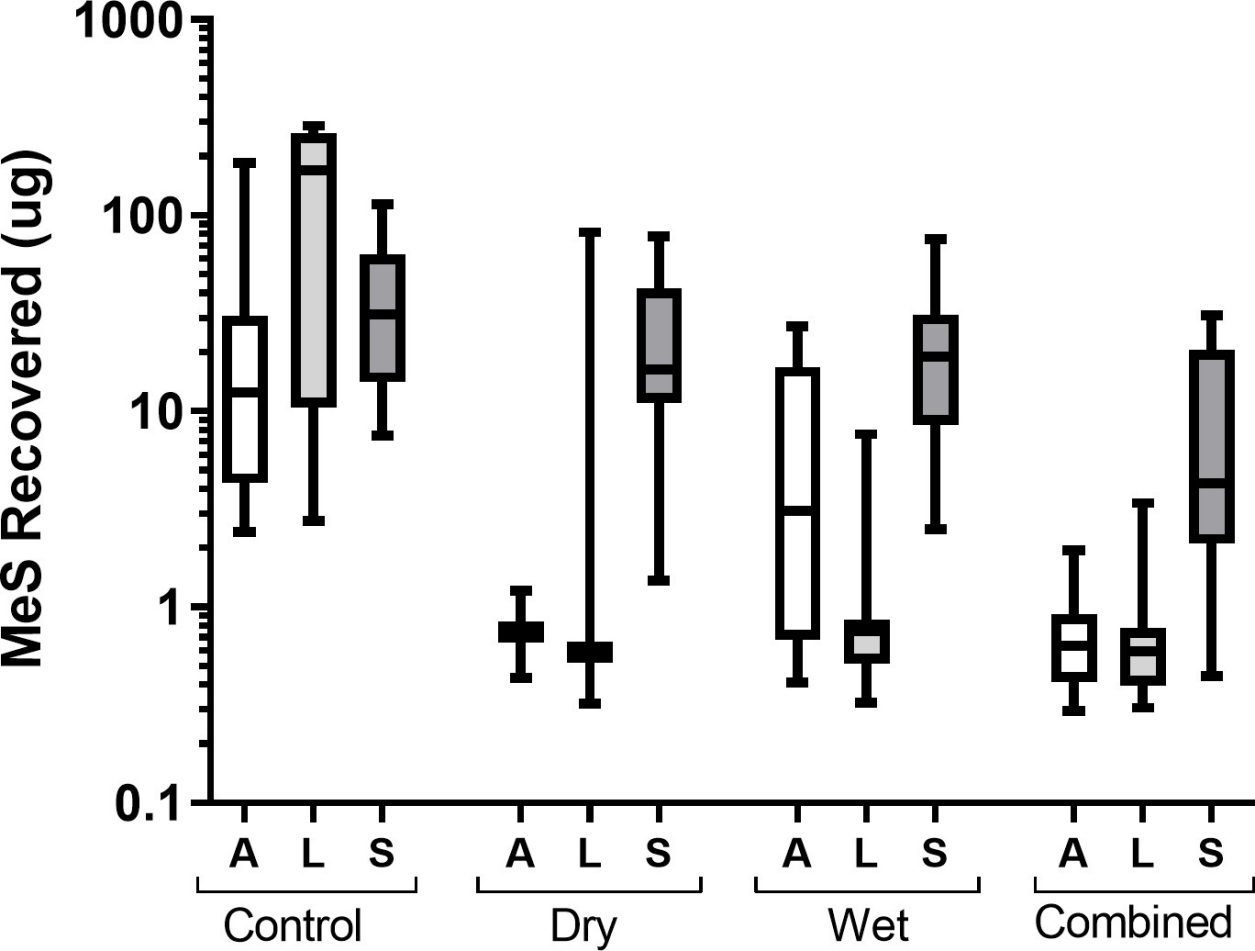
Total MeS recovered in skin samples for each application site in each decontamination condition. Box and whisker plot shows median and inter–quartile range, together with the maximum and minimum values. A = arm, L = leg, S = shoulder.

Not all application sites were decontaminated equally. Across conditions B, C and D, there was a significant main-effect of application site, *F*(2,22) = 10.37, *p* = .002, and no significant interaction between application site and decontamination condition, *F*(4,44) = 1.93, *p* = .139. Although there was no significant difference in MeS recovered between the arms and legs (mean total recoveries ranged between 0.77–7.4 μg and 0.80–7.41 μg for arms and legs, respectively) *F*(1,11) < 0.01, *p* = .934, for all conditions, the shoulder showed a consistently higher recovery of MeS (range 9.65–26.17 μg), *F*(1,11) = 12.86, *p* = .004 (Fig 6 and S3 Table), supported by the UV imaging. Within the control condition, there was also a significant main-effect of application site, *F*(2,22) = 13.58, *p* = .002, although the pattern of distribution of simulant across application sites differed from the active decontamination conditions. Pairwise comparisons found that significantly more MeS was recovered from the legs than both the arms and shoulders (both *p* < .01), but there was no significant difference in MeS recovered between the arm and shoulder (*p* = .346).

### Correlation between amount of white roll and dry decontamination efficacy

**Fig 7:**
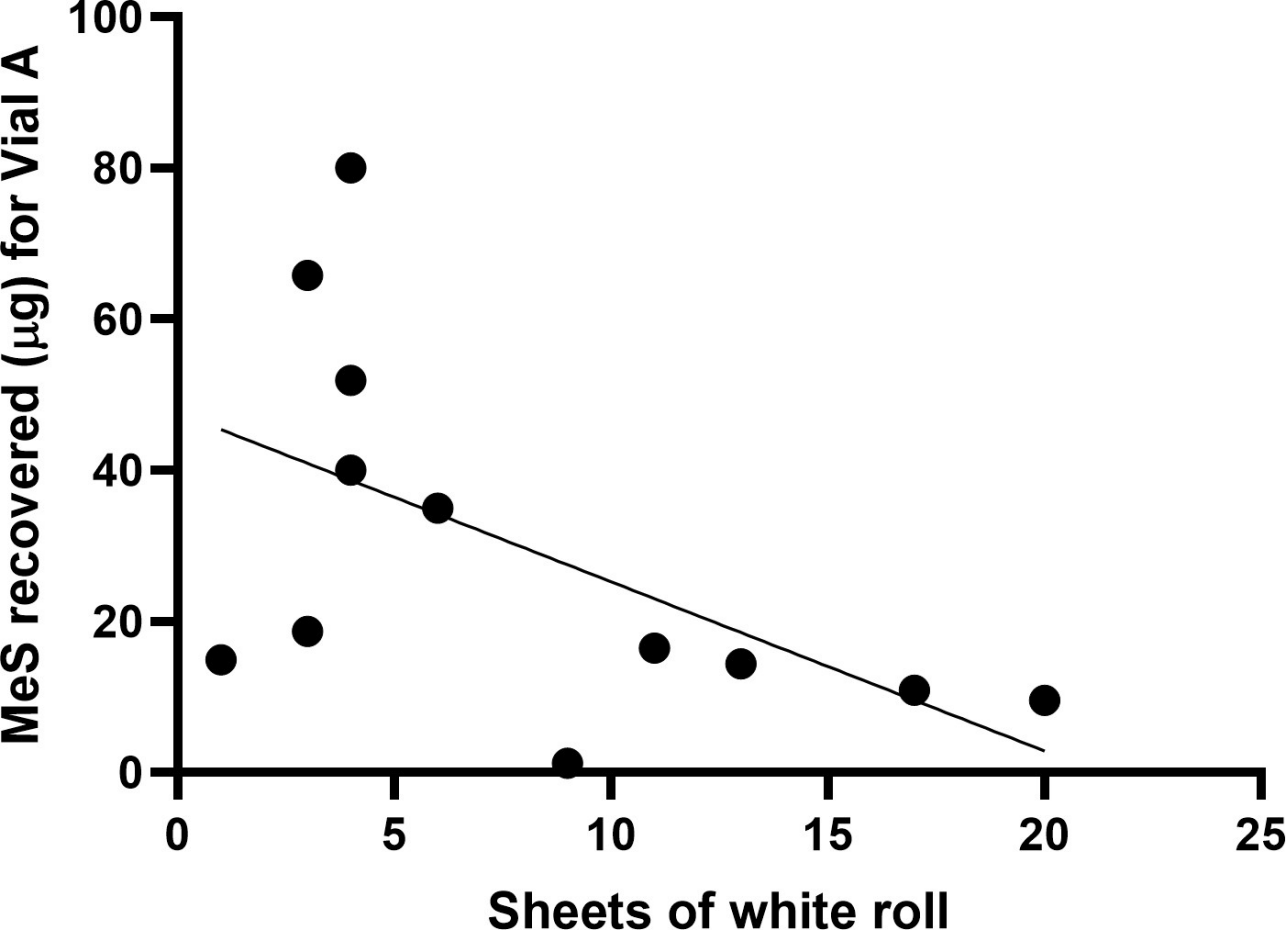
Correlation between number of sheets of white roll used as MeS recovered from skin in the dry decontamination condition (B). r_s_ = -.614, p = .034.

The number of pieces of white roll utilised by each volunteer during dry decontamination ranged between 1 and 20. A higher number of sheets of white-roll used during dry decontamination was significantly associated with lower total MeS recovered within both the dry and combined decontamination conditions, r_s_ = -.614, p = .034 for dry condition, r_s_ = -.645, p = .032 for combined condition (Fig 7). Correlations between MeS recovered and time spent on dry decontamination are not reported, due to high amount of missing data.

### Urinalysis

**Fig 8:**
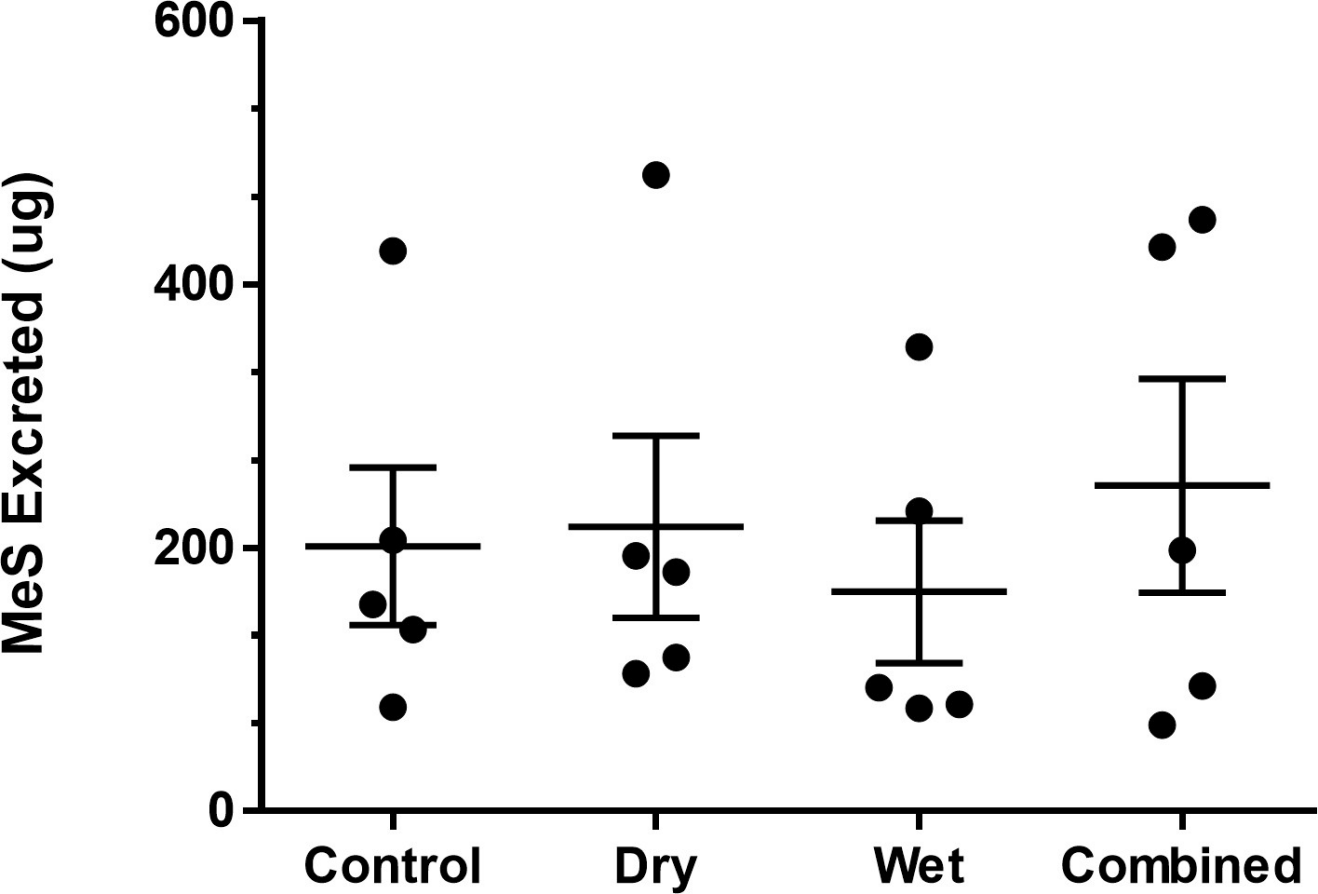
Total MeS excreted in urine over 24 hours by each participant in each decontamination condition, along with mean and *SE* in each condition.

Urine sample data was excluded from the analysis when participants had missed one or more samples (n = 7). Despite MeS being detected above the LOQ (4.6 ng/mL) in every urine sample analysed, there was no significant main-effect of decontamination condition on MeS excreted in urine, *F*(3,12) = 0.87, *p* = .467, and no significant difference between the active decontamination conditions and the no decontamination control, *F*(1,4) = 0.09, *p* = .774 (see Fig 8). Negligible excretion (indistinguishable above baseline levels) of MeS was observed in samples provided 8h and over after simulant application. This could relate to the urinary half-life of MeS, and future studies should consider not collecting samples after 8h post simulant application to increase protocol adherence by volunteers.

## Discussion

This study investigated the effectiveness of improvised dry and wet decontamination protocols, conducted alone and in combination, for decontaminating skin during chemical incidents. This study is the first to our knowledge to employ a cross-over design to reduce variability, and thus improve sensitivity for comparing the efficacy of different decontamination protocols. MeS was recovered from the skin of all volunteers at quantifiable levels (up to 24.4% of the applied dose), representing a substantial improvement compared to previous studies that only recovered a mean of 0.6% from application sites in control conditions [14]. Similar previous human volunteer studies such as the evaluation of the US Federal decontamination guidelines [15] have shown high variability in MeS recovery, albeit under field trial conditions, including only 0.002% recovery of MeS from an application zone in a control condition.

Results from both MeS quantitation and UV imaging demonstrated that there was less simulant remaining on the skin following each of the improvised decontamination protocols compared to no-decontamination control, consistent with previous in vitro and human volunteer studies [3–7]. In addition, more simulant was removed from the skin when both improvised dry and wet decontamination were conducted in sequence compared to individually. Thus, these findings provide evidence that both dry and wet improvised decontamination procedures are effective interventions for reducing the presence of contaminants on the skin surface and there is a benefit to conducting dry and wet decontamination in combination. It cannot be ruled out however that continuing either dry or wet improvised decontamination to cover the additional time spent upon combined decontamination may have a comparable beneficial effect; this also may be a pragmatic approach depending on what resources are immediately available. Further studies should investigate this possibility. It should also be noted that in the combined decontamination condition there was a slight delay (3 minutes) to allow time for image capture between participants finishing dry decontamination and beginning wet decontamination. There is the potential that the combined intervention would have been more efficacious should the two procedures be conducted without a delay, which requires further investigation.

Following decontamination, there was more simulant remaining on the shoulders compared to both the arms and the legs. This finding supports our initial hypothesis that improvised decontamination procedures are less effective for less accessible areas of the body. One solution to this problem in practice may be to use ‘buddy systems’, where casualties assist each other with the decontamination process [16]. Interim and specialist decontamination showers, which are expected to be available to casualties at later stages in the incident response, may also be more effective at removing contaminants from more difficult to reach areas of the body.

During dry decontamination, participants were free to use as many or few pieces of dry material as they felt was required to decontaminate themselves. It was found that using more pieces of dry material was associated with less simulant contaminant remaining on the skin, both when dry decontamination was conducted alone and in sequence with wet decontamination. This finding raises the possibility that using more dry material may result in more effective decontamination. However, further research controlling the quantity of dry material used in decontamination is required to demonstrate a causal effect on decontamination efficacy. Further research should also seek to establish optimal quantities of dry decontamination material, allowing some pre-planning for the provision of sufficient clean, dry material in operational settings. It is important to note however, that a pragmatic approach to improvised decontamination should be taken; as well as bringing resources to the scene, responders should make use of any available clean, dry and absorbent material available in the immediate area.

Due to missed samples, several participants’ urine data was excluded from analysis, resulting in a low sample size. Nonetheless, unexpectedly, no significant differences between MeS concentrations excreted in urine across control and intervention conditions was observed. These findings are consistent with a recent report from Larner *et al* [7] which reported no significant differences in the concentrations of urinary salicyluric acid (a metabolite of MeS) in volunteers contaminated with MeS in a similar decontamination study. There are several potential reasons for this. One possibility is that dietary sources of MeS are significant and mask the contribution of the dermal MeS dose to urinary MeS and salicyluric acid. Although participants were instructed to avoid products containing MeS for 24 hours prior to each session, MeS is widely prevalent in dietary products (e.g. [17]). Dietary sources of MeS would need to be more tightly controlled to rule out the possibility of masking dermal contributions. Another possibility is that MeS skin absorption is rapid and the 15-minute delay before commencing intervention is insufficient to influence systemic levels significantly. When MeS was examined in urine in a feasibility study using 100uL applied simulant [10] peak urine levels at 1h were significantly higher than baseline but other time points did not reach significance. Further examination of suitable MeS application dose and how to avoid MeS exposure from dietary and other sources is required. In addition, alternative simulant contaminants should be explored which may be more suitable for assessing systemic exposure in urine [10].

The findings of this study provide support for continued inclusion of improvised decontamination in IOR. Although improvised decontamination did not completely remove the contaminant, by starting the decontamination process as early as possible, continued exposure to harmful chemicals and associated risks are reduced. In addition, the clear demonstration of substance removal from skin demonstrates that improvised decontamination can effectively improve protection of non-exposed bystanders and responders from accidental cross-contamination.

Evidence supporting the effectiveness of improvised decontamination indicates that it would be beneficial to increase awareness of these procedures in the general public and people employed in areas at high risk of a chemical incident. Since improvised decontamination can be conducted using widely available resources, these procedures could in principal be performed without instruction from emergency responders, provided members of the public have sufficient prior knowledge of the procedures. Eliminating the need to wait for emergency responders to arrive at a scene would enable the decontamination process to begin sooner, further reducing exposure and risk of injury. Previous research has demonstrated that a public information campaign to raise awareness of initial decontamination actions was perceived as both useful and beneficial by the public [18].

One limitation of the present study, is that participants conducted decontamination following one-on-one instruction from a researcher under well controlled and well observed conditions. Although decontamination efficacy was good in this study, in a mass casualty incident, it will be more difficult for emergency responders to communicate decontamination instructions and monitor compliance, which may in turn result in less thorough and effective decontamination. Previous research has demonstrated that the effectiveness of dry decontamination can be dependent on both the provision of sufficient guidance from emergency responders and casualty compliance with instructions provided [5, 6]. Another limitation is the extent to which these findings can be generalised for all chemicals. This study used MeS, a simulant for sulphur mustard which has been widely used in previous studies [8]. The effectiveness of decontamination procedures may vary for contaminants with different physicochemical properties. This study did not find any evidence of a significant difference in effectiveness between wet and dry procedures, despite MeS being noticeably hydrophobic, but it is possible that wet and dry procedures may differ in their effectiveness for other chemicals. Further work is required to determine the effectiveness of different decontamination procedures for contaminants with different physicochemical characteristics. We have recently identified benzyl salicylate (BeS) as a novel simulant for less volatile and more persistent chemical threats [19]. Consideration should be given to evaluation of the decontamination methods tested here with this new simulant. A third limitation of this study is that human volunteer studies can only measure the quantity of simulant in surface layers of the skin following decontamination. In vitro studies will be required to compare the effects of decontamination protocols on the presence of simulant deeper within the skin.

This study clearly indicates that both wet and dry improvised decontamination procedures can be effective means of rapidly removing contaminants from the skin, and that there is a cumulative benefit to conducting dry and wet improvised decontamination in sequence. However, improvised procedures do not guarantee the complete removal of contaminants, particularly from areas of the body that are more difficult for casualties to reach. Improvised decontamination should therefore be followed-up with more comprehensive procedures, such as interim and specialist decontamination showers, as currently recommended in both UK and US operational response guidance [1, 20] nonetheless, by beginning to remove contaminants from the skin as soon as possible, improvised decontamination procedures can limit exposure to harmful materials and reduce the risk of injury in chemical incidents.

## Conclusions

Improvised dry and wet decontamination protocols are effective at rapidly reducing risk to casualties–providing important new evidence to support their continued inclusion within current UK Initial Operational Response. When conducted in sequence, the decontamination protocols were significantly more effective at removing simulant from skin compared to just dry and wet decontamination alone, although further work is required to determine if performing dry or wet decontamination alone for an extended period, until the arrival of specialist resources, would be just as or more beneficial. The effectiveness of dry decontamination, either individually or in combination with wet decontamination, was associated with the use of more dry material. The analysis of MeS in urine was problematic and inconclusive and further work is required to improve the measurement of this simulant in urine as a marker of systemic exposure.

## Supporting information

S1 Fig Mean (and SE) area of fluorescence over time for each decontamination condition. UV2 = post-application (T13), UV3 = post-decontamination 1 (T19), UV4 = post-decontamination 2 (T25).
(TIF)

S2 Fig Mean (and SE) fluorescent emittance over time for each decontamination condition. UV2 = post-application (T13), UV3 = post-decontamination 1 (T19), UV4 = post-decontamination 2 (T25).
(TIF)

S3 Fig Total recovery of MeS from skin samples after each decontamination condition. Box and whisker plot shows median and inter–quartile range, together with the maximum and minimum values. A, B and C represent the skin sampling vials containing disks 1+2, 3+4 and 5+6, respectively.
(TIF)

S1 Table Mean (*SD*) area of fluorescence (cm²) for each application site in each decontamination condition. (PDF)

S2 Table Mean (*SD*) fluorescent emittance index for each application site in each decontamination condition. (PDF)

S3 Table Mean (*SD*) total amount of MeS (μg) recovered from skin for each application site in each decontamination condition. (PDF)
