# Supplementary figures and images for: A controlled cross-over study to evaluate the efficacy of improvised dry and wet emergency decontamination protocols for chemical incidents
Source: PLoS One. 2020 Nov 4;15(11):e0239845. doi: 10.1371/journal.pone.0239845 (PMC7641342; doi:10.1371/journal.pone.0239845)

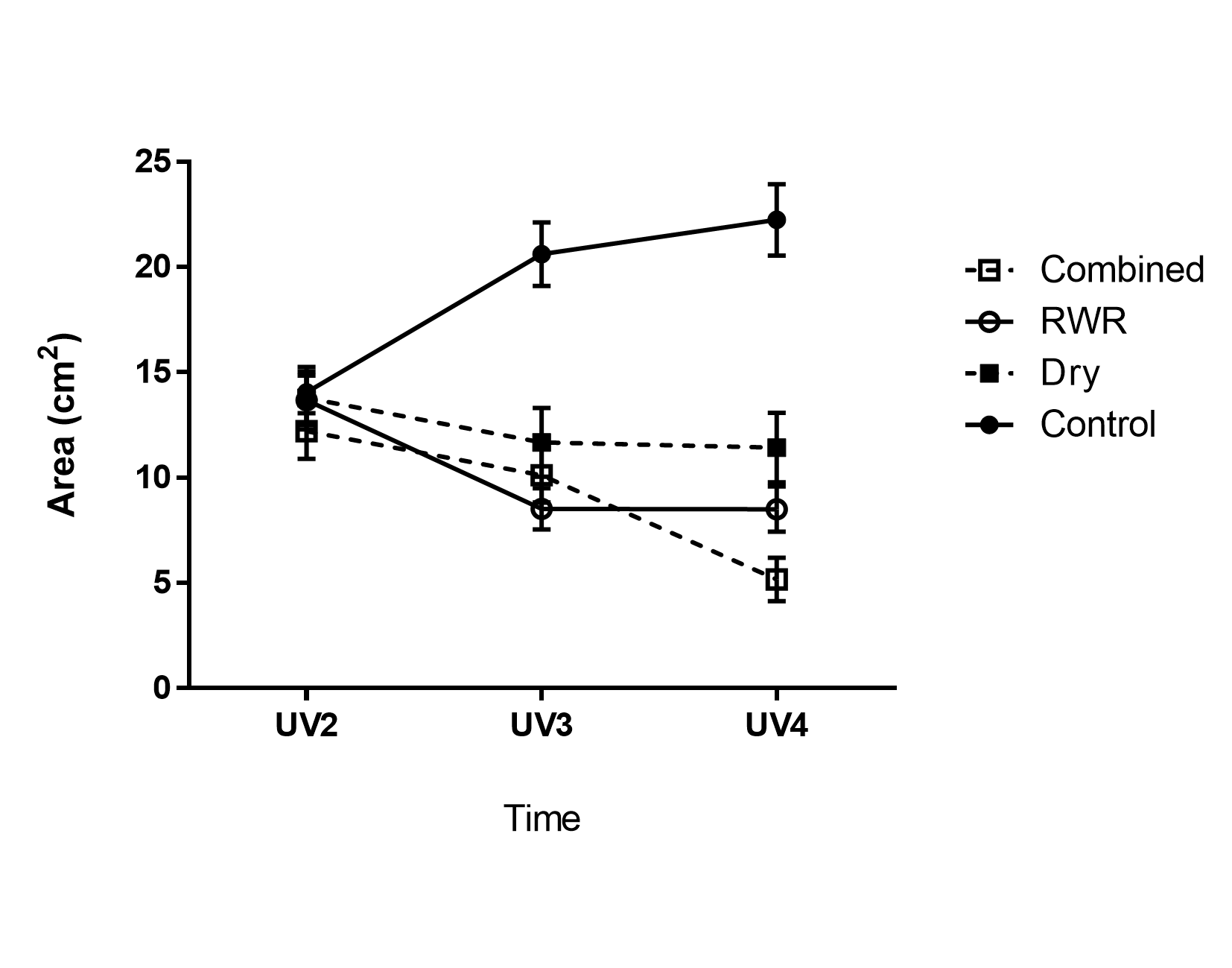

Supplement: S1 Fig — UV2 = post-application (T13), UV3 = post-decontamination 1 (T19), UV4 = post-decontamination 2 (T25). (TIF) [file pone.0239845.s001.tif]

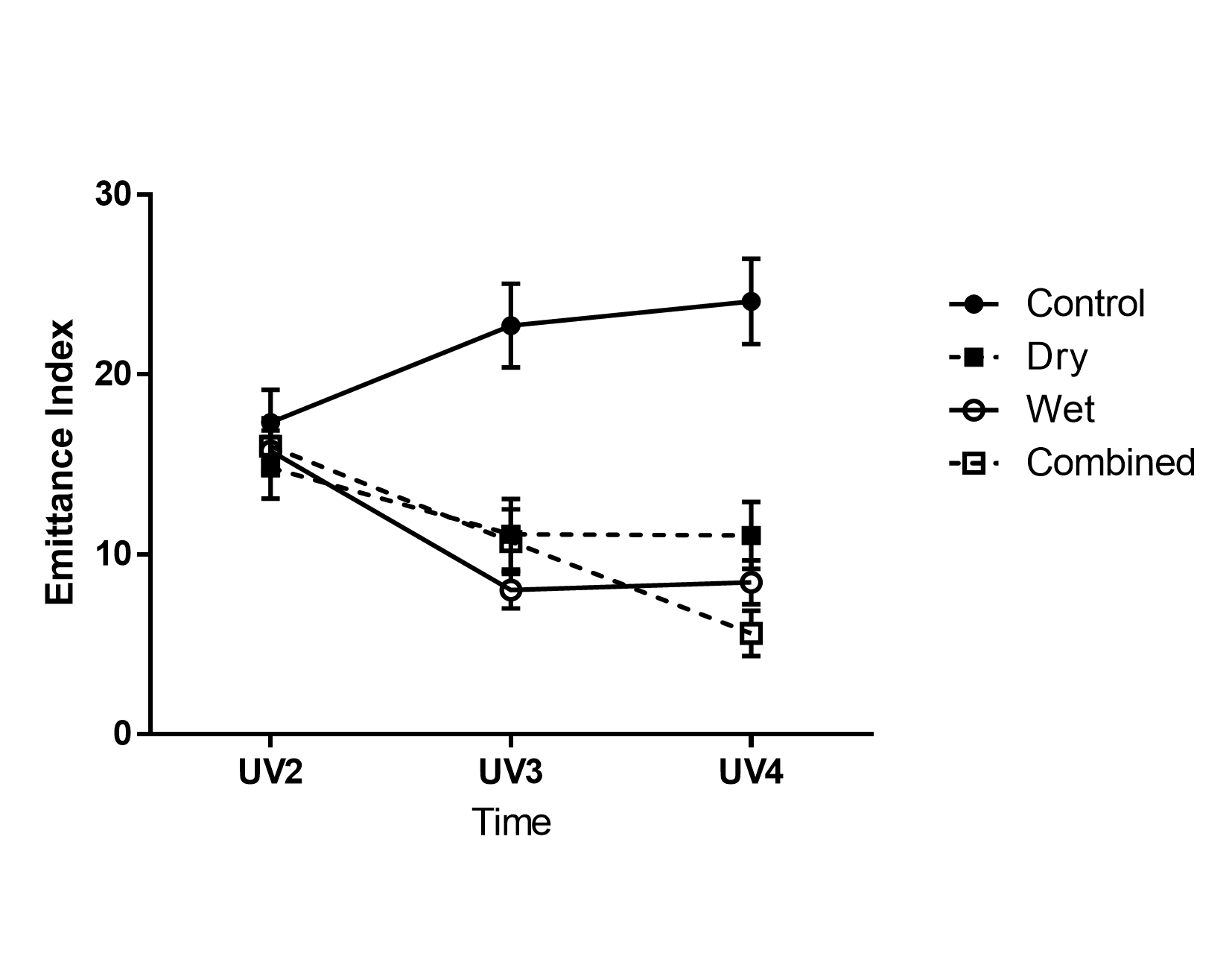

Supplement: S2 Fig — UV2 = post-application (T13), UV3 = post-decontamination 1 (T19), UV4 = post-decontamination 2 (T25). (TIF) [file pone.0239845.s002.tif]

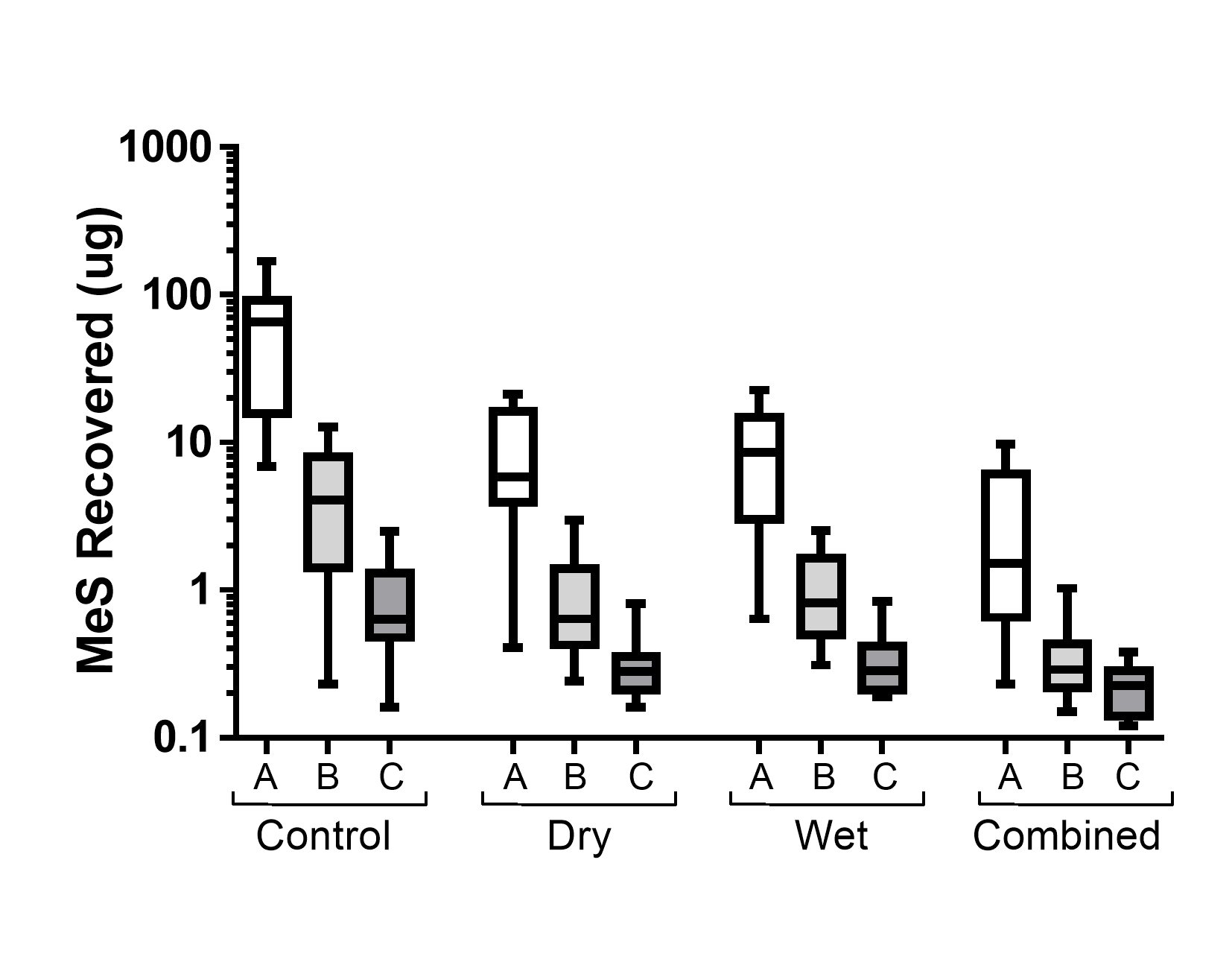

Supplement: S3 Fig — Box and whisker plot shows median and inter–quartile range, together with the maximum and minimum values. A, B and C represent the skin sampling vials containing disks 1+2, 3+4 and 5+6, respectively. (TIF) [file pone.0239845.s003.tif]
