# Supplementary material for: A controlled cross-over study to evaluate the efficacy of improvised dry and wet emergency decontamination protocols for chemical incidents
Source: PLoS One. 2020 Nov 4;15(11):e0239845. doi: 10.1371/journal.pone.0239845 (PMC7641342; doi:10.1371/journal.pone.0239845)
Supplement: S1 Table — (PDF) [file pone.0239845.s004.pdf]

S1 Table. Mean (*SD*) area of fluorescence (cm^2) for each application site in each decontamination condition.

		Decontamination Condition			
		A – Control	B – Dry	C – Wet	D – Combined
**Image**	**Application Site**	(*N* = 12)	(*N* = 12)	(*N* = 12)	(*N* = 12)
UV2	Arm	6.00 *(2.01)*	5.53 *(2.42)*	5.69 *(1.99)*	5.11 *(2.35)*
	Leg	2.74 *(1.44)*	2.99 *(1.78)*	2.69 *(0.98)*	2.53 *(0.76)*
	Shoulder	5.31 *(1.36)*	5.26 *(2.06)*	5.31 *(2.07)*	4.57 *(1.97)*
	**Total**	**14.05 *(3.44)***	**13.78 *(5.14)***	**13.68 *(4.06)***	**12.21 *(4.54)***
UV3	Arm	8.05 *(2.03)*	3.68 *(2.74)*	2.11 *(1.59)*	3.16 *(2.66)*
	Leg	4.32 *(2.44)*	1.35 *(1.86)*	1.10 *(1.34)*	1.35 *(1.66)*
	Shoulder	8.24 *(2.33)*	6.66 *(3.25)*	5.31 *(1.74)*	5.60 *(2.21)*
	**Total**	**20.61 *(5.21)***	**11.68 *(5.66)***	**8.52 *(3.40)***	**10.11 *(4.46)***
UV4	Arm	8.42 *(2.22)*	3.05 *(2.40)*	1.92 *(1.70)*	0.61 *(1.02)*
	Leg	4.79 *(2.67)*	1.38 *(1.99)*	0.99 *(1.16)*	0.33 *(0.83)*
	Shoulder	9.02 *(2.68)*	6.99 *(3.54)*	5.60 *(2.26)*	4.23 *(2.84)*
	**Total**	**22.24 *(5.86)***	**11.43 *(5.72)***	**8.51 *(3.71)***	**5.17 *(3.55)***
UV5	Arm	8.48 *(2.22)*	3.16 *(2.20)*	1.89 *(1.82)*	0.53 *(0.92)*
	Leg	4.95 *(2.69)*	1.46 *(1.99)*	0.89 *(1.09)*	0.21 *(0.62)*
	Shoulder	9.21 *(2.88)*	7.08 *(3.51)*	5.55 *(1.95)*	4.14 *(2.83)*
	**Total**	**22.64 *(6.29)***	**11.70 *(5.25)***	**8.32 *(3.34)***	**4.87 *(3.38)***
UV6	Arm	5.60 *(1.56)*	2.14 *(1.73)*	1.08 *(1.21)*	0.33 *(0.50)*
	Leg	4.18 *(2.28)*	1.02 *(1.46)*	0.77 *(1.07)*	0.18 *(0.52)*
	Shoulder	6.32 *(2.65)*	5.10 *(2.87)*	3.76 *(1.56)*	2.88 *(2.28)*
	**Total**	**16.10 *(5.10)***	**8.26 *(4.19)***	**5.61 *(2.42)***	**3.39 *(2.60)***
