# Supplementary material for: A controlled cross-over study to evaluate the efficacy of improvised dry and wet emergency decontamination protocols for chemical incidents
Source: PLoS One. 2020 Nov 4;15(11):e0239845. doi: 10.1371/journal.pone.0239845 (PMC7641342; doi:10.1371/journal.pone.0239845)
Supplement: S2 Table — (PDF) [file pone.0239845.s005.pdf]

S2 Table. Mean (*SD*) fluorescent emittance index for each application site in each decontamination condition.

		Decontamination Condition			
		A – Control	B – Dry	C – Wet	D – Combined
**Image**	**Application Site**	(*N* = 12)	(*N* = 12)	(*N* = 12)	(*N* = 12)
UV2	Arm	7.29 *(2.84)*	6.07 *(2.59)*	6.41 *(1.54)*	6.74 *(2.84)*
	Leg	2.87 *(1.10)*	2.81 *(1.50)*	2.69 *(0.86)*	3.03 *(0.96)*
	Shoulder	7.17 *(3.44)*	5.93 *(2.82)*	6.66 *(2.56)*	6.25 *(2.65)*
	**Total**	**17.33 *(6.36)***	**14.81 *(5.94)***	**15.76 *(3.92)***	**16.02 *(5.42)***
UV3	Arm	8.85 *(3.59)*	3.08 *(3.05)*	1.94 *(2.01)*	3.28 *(3.60)*
	Leg	3.72 *(1.34)*	0.85 *(1.20)*	0.72 *(0.94)*	1.05 *(1.76)*
	Shoulder	10.16 *(4.53)*	7.19 *(4.27)*	5.37 *(2.05)*	6.38 *(2.90)*
	**Total**	**22.72 *(8.12)***	**11.12 *(6.84)***	**8.03 *(3.56)***	**10.71 *(6.26)***
UV4	Arm	9.26 *(3.93)*	2.49 *(2.29)*	1.91 *(2.23)*	0.67 *(1.12)*
	Leg	3.99 *(1.49)*	0.84 *(1.25)*	0.67 *(0.88)*	0.32 *(0.93)*
	Shoulder	10.81 *(4.36)*	7.71 *(4.63)*	5.88 *(2.68)*	4.63 *(3.32)*
	**Total**	**24.06 *(8.28)***	**11.05 *(6.47)***	**8.46 *(4.23)***	**5.61 *(4.40)***
UV5	Arm	8.87 *(3.81)*	2.35 *(1.85)*	1.84 *(2.23)*	0.57 *(1.00)*
	Leg	3.99 *(1.63)*	0.91 *(1.32)*	0.57 *(0.77)*	0.20 *(0.63)*
	Shoulder	10.70 *(4.36)*	7.89 *(4.80)*	5.73 *(2.14)*	4.55 *(3.44)*
	**Total**	**23.56 *(8.38)***	**11.15 *(6.27)***	**8.13 *(3.58)***	**5.31 *(4.22)***
UV6	Arm	5.86 *(2.63)*	1.64 *(1.48)*	1.14 *(1.59)*	0.37 *(0.57)*
	Leg	3.36 *(1.42)*	0.65 *(1.01)*	0.51 *(0.77)*	0.18 *(0.56)*
	Shoulder	7.51 *(3.63)*	5.65 *(3.54)*	3.89 *(1.81)*	3.52 *(2.96)*
	**Total**	**16.74 *(6.27)***	**7.94 *(4.54)***	**5.54 *(2.62)***	**4.06 *(3.38)***
