# Supplementary material for: A controlled cross-over study to evaluate the efficacy of improvised dry and wet emergency decontamination protocols for chemical incidents
Source: PLoS One. 2020 Nov 4;15(11):e0239845. doi: 10.1371/journal.pone.0239845 (PMC7641342; doi:10.1371/journal.pone.0239845)
Supplement: S3 Table — (PDF) [file pone.0239845.s006.pdf]

S3 Table. Mean (*SD*) total amount of MeS (μg) recovered from skin for each application site in each decontamination condition.

		Decontamination Condition			
**Application Site**	**Sample Vial**	A – Control (*N* = 12)	B – Dry (*N* = 12)	C – Wet (*N* = 12)	D – Combined (*N* = 12)
Arm	Vial A	29.82 *(50.37)*	0.42 *(0.12)*	6.90 *(9.19)*	0.44 *(0.42)*
	Vial B	0.67 *(0.89)*	0.17 *(0.05)*	0.30 *(0.27)*	0.17 *(0.06)*
	Vial C	0.29 *(0.11)*	0.17 *(0.07)*	0.20 (*0.09*)	0.15 (*0.07*)
	**Total**	**30.77 *(51.30)***	**0.76 *(0.20)***	**7.40 *(9.48)***	**0.77 *(0.49)***
Leg	Vial A	136.29 (*108.09)*	6.60 *(21.97)*	0.83 *(1.42)*	0.37 *(0.58)*
	Vial B	11.05 *(10.51)*	0.60 *(1.43)*	0.38 *(0.51)*	0.23 *(0.16)*
	Vial C	1.84 *(1.75)*	0.21 *(0.12)*	0.21 (*0.16*)	0.21 (*0.12*)
	**Total**	**149.18 *(116.39)***	**7.41 *(23.51)***	**1.41 *(2.07)***	**0.80 *(0.84)***
Shoulder	Vial A	37.29 *(30.24)*	22.90 *(20.19)*	21.71 *(21.13)*	8.50 *(10.32)*
	Vial B	2.38 *(1.66)*	2.63 *(2.97)*	2.75 *(2.29)*	0.83 *(0.89)*
	Vial C	0.66 *(0.55)*	0.63 *(0.68)*	0.73 (*0.64*)	0.32 (*0.19*)
	**Total**	**40.34 *(31.13)***	**26.17 *(23.58)***	**25.19 *(23.68)***	**9.65 *(11.16)***
**Total**		**220.29 *(168.81)***	**34.33 *(27.95)***	**34.00 *(25.09)***	**11.22 *(11.28)***
